# Supplementary material for: Cell-Type-Specific Signalling Networks Impacted by Prostate Epithelial-Stromal Intercellular Communication
Source: Cancers (Basel). 2023 Jan 23;15(3):699. doi: 10.3390/cancers15030699 (PMC9913520; doi:10.3390/cancers15030699)
Supplement: Supplementary file 1 [file cancers-15-00699-s001.zip › Cancers_Supplementary Figure S1_revised.docx]

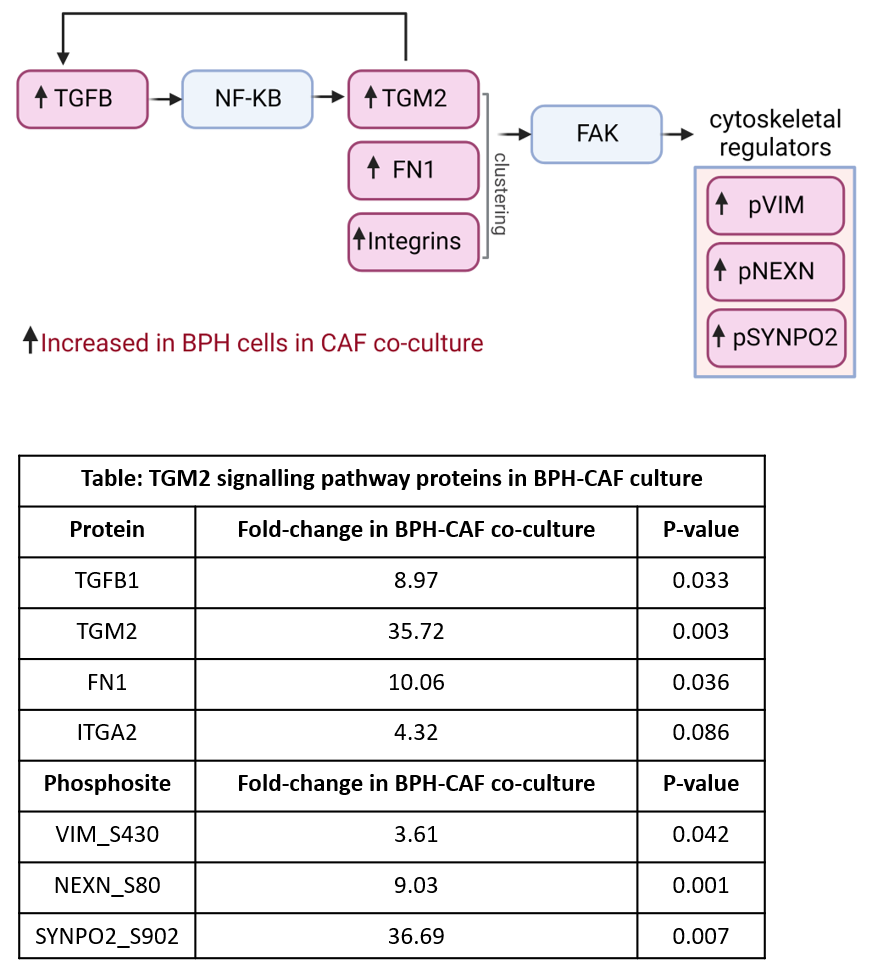


Supplementary Figure S1: **Changes in TGM2 signalling pathways in BPH-1 cells following CAF co-culture.** Known interactors up- and downstream of TGM2, reported by Eckert et al., 2014 [21] and Tatsukawa et al., 2016 [22], were investigated in the CTAP-MS analysis of BPH-1 cells in monoculture and CAF co-culture. BPH-1 proteins with increased fold-change in CAF co-culture are highlighted in pink, and listed in the associated table. Data displayed as mean fold-change of raw protein intensity from mass spectrometry measurement from three biological replicates and P-value calculated from Student’s t-test comparing monoculture and co-culture.
